# Supplementary material for: Picosecond time-resolved photon antibunching measures nanoscale exciton motion and the true number of chromophores
Source: Nat Commun. 2021 Feb 26;12:1327. doi: 10.1038/s41467-021-21474-z (PMC7910429; doi:10.1038/s41467-021-21474-z)
Supplement: Supplementary file 1 — Supplementary Information [file 41467_2021_21474_MOESM1_ESM.pdf]

Supplementary Information for

Picosecond time-resolved photon antibunching measures nanoscale exciton motion and the true number of chromophores

Gordon J. Hedley[1,†,], Tim Schröder[2, †], Florian Steiner[2], Theresa Eder[3], Felix Hofmann[3], Sebastian Bange[3], Dirk Laux,[4] Sigurd Höger,[4] Philip Tinnefeld[2], John M. Lupton[3] and Jan Vogelsang[3,*]*

[1]School of Chemistry, University of Glasgow, University Avenue, Glasgow, G12 8QQ, United Kingdom

[2]Department Chemie and Center for NanoScience (CeNS), Ludwig-Maximilians-Universität München, Butenandtstraße 5-13 Haus E, 81377 München, Germany

[3]Institut für Experimentelle und Angewandte Physik, Universität Regensburg, Universitätsstraße 31, 93053 Regensburg, Germany

[4]Kekulé-Institut für Organische Chemie und Biochemie, Universität Bonn, Gerhard-Domagk-Strasse 1, 53121 Bonn, Germany

Supplementary Methods

1. Details of DNA origami structures and sample preparation

The DNA origami structure[1] was modified using caDNAno (version 0.2.2, design schematics in Supplementary Figure 1). The scaffold is an 8064 nucleotide long ssDNA extracted from M13mp18 bacteriophages. All staple strands were purchased from Eurofins Genomics GmbH as well as the dye labeled oligonucleotides (see at the end of Supplementary Information). The ATTO 542 modified oligonucleotides for external labeling were purchased from biomers.net. Scaffold and oligonucleotides were mixed according to Supplementary Table1 for origami folding. The folding buffer (FB) is a Tris-EDTA buffer (1x TE, 10 mM Tris-HCl, 1 mM EDTA•Na_2) with 20 mM $MgCl_2$ and 5 mM NaCl. In the annealing process, the mixture was heated and slowly cooled down with a nonlinear thermal ramp over 16 hours according to reference.[2] After annealing, the excess staples were removed with polyethylene glycol (PEG) precipitation. The samples were mixed with an equal volume of PEG precipitation buffer (1× TAE, 15 % (w/v) PEG-8000, 500 mM NaCl, 12 mM $MgCl_2$) and centrifuged at 16 krcf (thousand relative centrifugal force, i.e. 1000 g) for 30 min at 4 °C. After removing the supernatant, the pellet was suspended in 1× FB. Afterwards, the DNA origami was externally labeled with ATTO 542 modified oligonucleotides. A threefold excess with respect to the extended staples was used and incubated for 20 min in a wet chamber at room temperature. The DNA origami structures were purified via gel electrophoresis. A 1.5 % agarose gel containing a Tris base, acetic acid and EDTA buffer (0.5× TAE, 20 mM Tris-HCl, 10 mM acetic acid, 0.5 mM EDTA) and 12 mM $MgCl_2$ was used at 60 V for 2 hours in a gel box cooled in an ice-water bath. The gel was not stained to avoid staining reagent-dye interactions. On a blue illuminated table DNA origami structures could be seen due to the numerous ATTO 542 dyes. DNA origami structures were recovered from the target band. The samples were stored at -26 °C until further use.

Folding Table

Final concentrations for DNA origami folding are given in Supplementary Table 1. The meaning of the reagents is described below:

Supplementary Table 1: Folding reagents with final concentrations.

Reagent	Final concentration / nM
scaffold	25
core staples	225
biotin staples	250
extended staples	225
dye and refill staples	225

scaffold: Single-stranded viral 8064 nt ssDNA from M13mp18.

core staples: Contains every unmodified staples of the rectangular DNA origami. The wildtype structure is given in reference[1].

biotin staples: Four biotin modified staples. Modifications are placed at the 3’ end.

extended staples: 13 staples extended at the 3’ end for external labeling. The extended sequence is: 5’ TTTTCCTCTACCACCTACATCAC 3’. Sequence for the ATTO542 oligonucleotides: 5’ GTGATGTAGGTGGTAGAGGA-ATTO542 3’

dye and refill staples: Oligonucleotides labeled with ATTO 647N at the 5’ end. For structures which do not need all five dyes the respective oligonucleotides were substituted with unmodified oligonucleotides.

Confirming successful DNA origami structure assembly with TEM imaging

Successful assembly of the modified DNA origami structure was confirmed with TEM imaging. The origami structures were imaged on Ar-plasma cleaned TEM grids (Formvar/carbon, 400 mesh, Cu, TedPella, Inc., USA). The DNA origami structures were stained with a 2 % uranyl formate solution. The imaging was performed on a JEM-1100 microscope (JEOL GmbH, Japan) with an acceleration voltage of 80 kV.

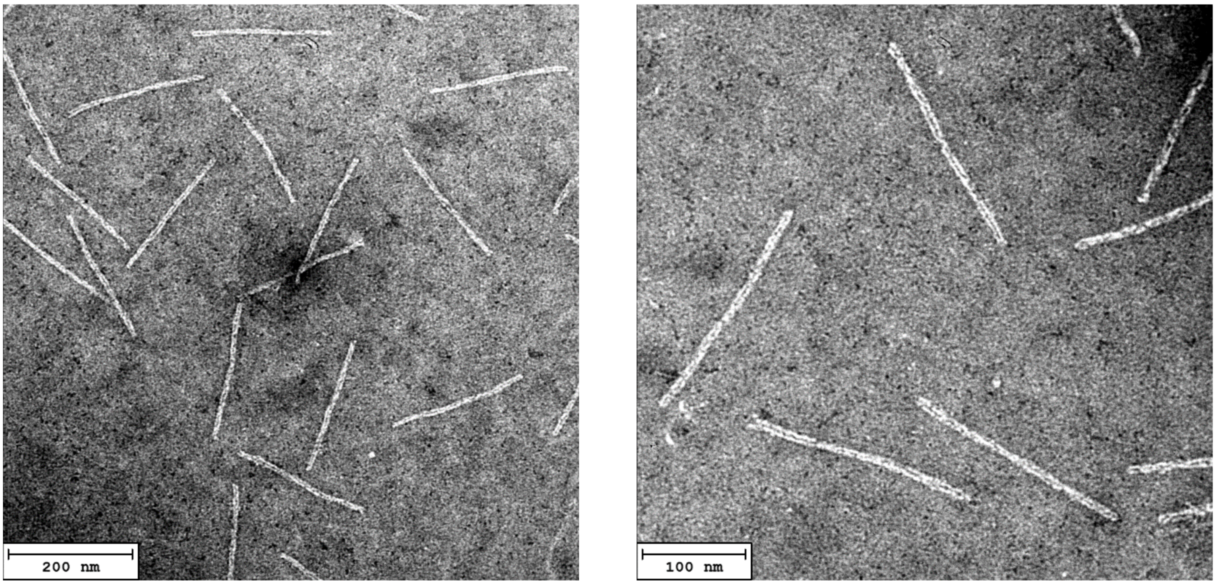

Supplementary Figure 1. *Two representative negative stain images of the modified DNA origami structure with different magnifications. The expected length of the DNA origami structure is 225 nm. The TEM images show successful assembly of the DNA origami structures. No nicks were found at the position which was modified for our needs and is located at the center of the structure (orange and blue staples in Supplementary Figure 2).*

Surface preparation and immobilization

Samples were measured in LabTek^{TM} chamber slides (Thermo Fisher Scientific Inc.) which were cleaned twice for 20 minutes with 0.1 M hydrofluoric acid (AppliChem GmbH) and washed afterwards three times with ultrapure water. For sample immobilization the glass surface was coated with biotin labeled bovine serum albumin (BSA) (1 mg/mL Sigma-Aldrich Chemie GmbH) and NeutrAvidin (1 mg/mL, Sigma-Aldrich Chemie GmbH). The DNA origami structures (60 pM in 1x FB) were immobilized by the biotin-NeutrAvidin binding.

Sample preparation for single-molecule measurements

Traces were recorded from immobilized DNA origami structures. Photo blinking and photo bleaching was reduced with an oxidizing and reducing buffer system (1× TAE, 12 mM $MgCl_2$, 2 mM Trolox/Troloxquinone, 1 % (w/v) D-(+)-Glycose) [3] in combination with an oxygen scavenging system [4] (1 mg mL^{-1} glucose oxidase, 0.4 % (v/v) catalase (50 µg mL^{-1}), 30 % glycerol, 12.5 mM KCl in 50 mM TRIS). The oxygen scavenging system was added to the oxidation and reducing buffer at a concentration of 10 % (v/v) in the LabTek™ system. DNA origami structures were excited with a laser power adjusted to 1 µW for all samples, except for the 5-dye sample which was measured with 0.5 µW to reduce spectral shifts and photo bleaching. The laser repetition rate was 40 MHz.

Traces for psTRAB data processing were recorded for 10 seconds. Origami structures with five dyes were recorded for 5 seconds.

DNA origami structure

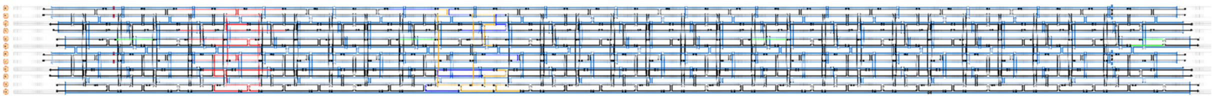

Supplementary Figure 2. *caDNAno sketch of the DNA origami structure used. Modified staples are colored. Green staples are biotin labeled. Red staples are extended at the 3' (5' TTTTCCTCTACCACCTACATCAC) end for external labeling with ATTO 542 modified oligonucleotides. Orange staples are labeled at the 5' end with ATTO 647N. Blue staples are next to the ATTO 647N labeled staples to stabilize the structure.*

psTRAB data processing

Every trace was evaluated and only traces with constant fluorescence signal and without photobleaching were used for further data processing. This is necessary because singlet-singlet-annihilation leads to highly excited states of the organic fluorophores which are prone to bleaching. Additionally, small spectral shifts were observed more frequently. In our analysis, we only used whole traces. A bleaching event changes the exciton-exciton-interaction, which contaminates the photon statistics simply because the number of physical emitters changes over time. Therefore, it is important that we only sum over traces which arise from the same number of physical emitters that give a constant fluorescence signal over the 10 seconds of recording.

2. PL transient of a five-dye sample

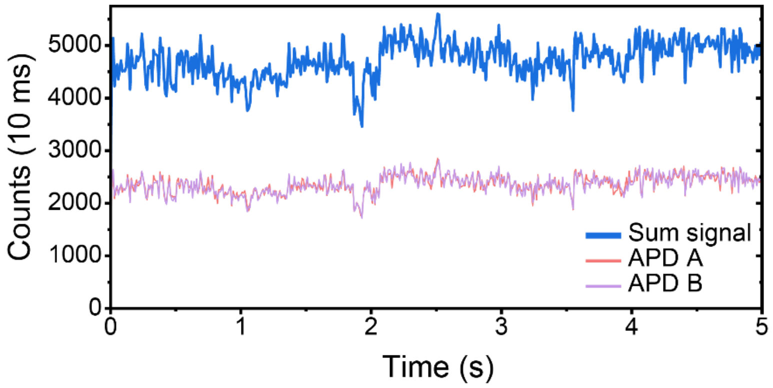

Supplementary Figure 3. *Representative PL trajectory of a five-dye origami sample with 10 ms binning. The blue trajectory is the sum signal of both detection channels which are shown in red and purple. All five dye trajectories were recorded for five seconds to prevent photodamage and spectral shifts.*

3. Instrument response function of the microscope for the DNA origami samples

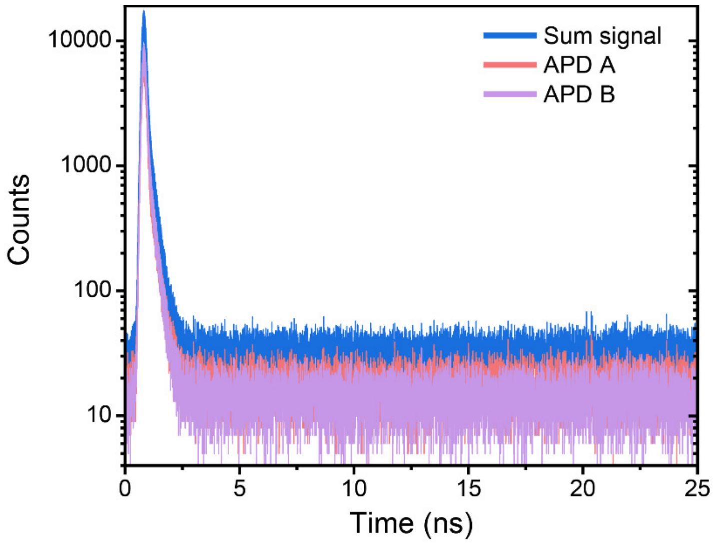

Supplementary Figure 4. *Instrument response function of the DNA origami setup with 4 ps binning. The microtime = 0 in figure 2b and 3 corresponds to the peak of the sum signal (blue).*

Supplementary Discussions

4. Number of independent emitters n as a function of SBR

For calculating the expected n for a single dye on the DNA origami setup the signal-to-background ratio (SBR) was calculated from a single-particle trajectory and a background trace from the same sample. Uncorrelated background adds correlation events equally to N_c and N_l. This raises the $N_\mathrm{c}/N_\mathrm{l}$ ratio and therefore a high SBR is desirable. Supplementary Figure 5a shows the average of the SBR ratios of both APDs. The microtime = 0 corresponds to the peak of the IRF as shown in Supplementary Figure 4. Due to scattering from the excitation laser pulse the SBR rises after the IRF. After reaching its maximum it decays due to the exponential fluorescence decay. The expected $N_\mathrm{c}/N_\mathrm{l}$ ratio was calculated according to the adapted relation from Weston *et al.* [5]:

$$\frac{N_\mathrm{c}}{N_\mathrm{l}} = \frac{1+\frac{S}{B}}{\left(1+\frac{S}{2B}\right)^2} \qquad \text{(Supplementary Equation 1)}$$

This relation gives the expected N_c/N_l ratio for a single independent emitter as a function of signal S and background B. For infinite SBR this value becomes zero.

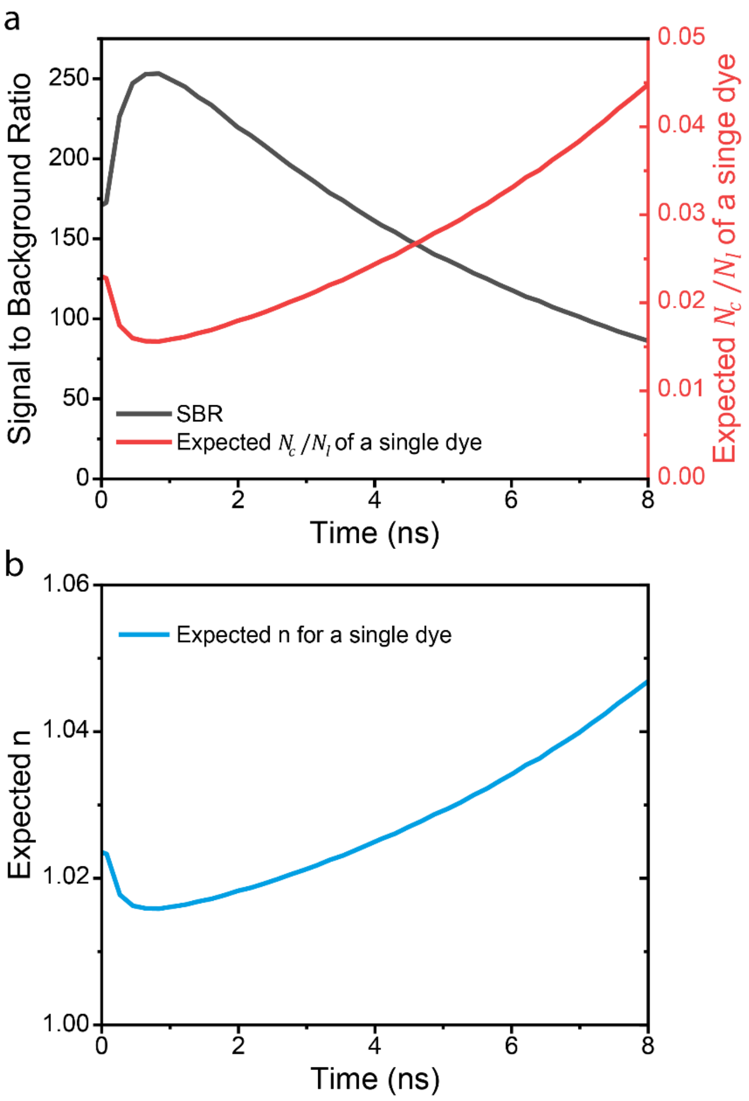

Supplementary Figure 5. *(a) Signal-to-background ratio of a single fluorescent dye in a DNA origami structure shown in black. Time = 0 corresponds to the peak of the IRF as shown in Supplementary Figure 4. The corresponding* N_c/N_l *ratio is shown in red. (b) Expected* n *for measured SBR in (a) according to Supplementary Equation 1. For infinite SBR this value becomes one.*

5. Complete fitting results of DNA origami structures

Supplementary Table 2: Complete fitting results with standard error of DNA origami structures according to eq. (3). Values in parentheses were fixed.

	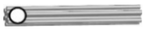	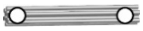	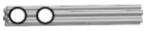	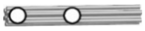	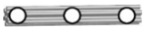	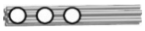	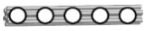
**y_0 (1)**	0.9821 ± 0.0017	0.5390 ± 0.0044	(0.9821)	(0.9821)	(0.9821)	(0.9821)	(0.9821)
**A_1 (1)**	(0)	0.0001 ± 0.0043	0.4094 ± 0.0057	0.414 ± 0.010	0.5948 ± 0.0064	0.351 ± 0.037	0.350 ± 0.037
**k_1** (ns^{-1})	(0)	-0.0078 ± 0.0048	1.718 ± 0.054	0.054 ± 0.0061	0.0628 ± 0.0033	1.44 ± 0.13	1.26 ± 0.12
**A_2 (1)**	(0)	(0)	(0)	(0)	(0)	0.264 ± 0.038	0.420 ± 0.038
**k_2** (ns^{-1})	(0)	(0)	(0)	(0)	(0)	0.356 ± 0.038	0.272 ± 0.021

6. Determination of the size of aggregates

Polymer chain numbers are estimated by counting spots in fluorescence images of samples before and after solvent vapour annealing (SVA). The starting concentration allows single polymer chains to be counted, as shown in Supplementary Figure 6, where we count the spots. This gives us the number of single chains.

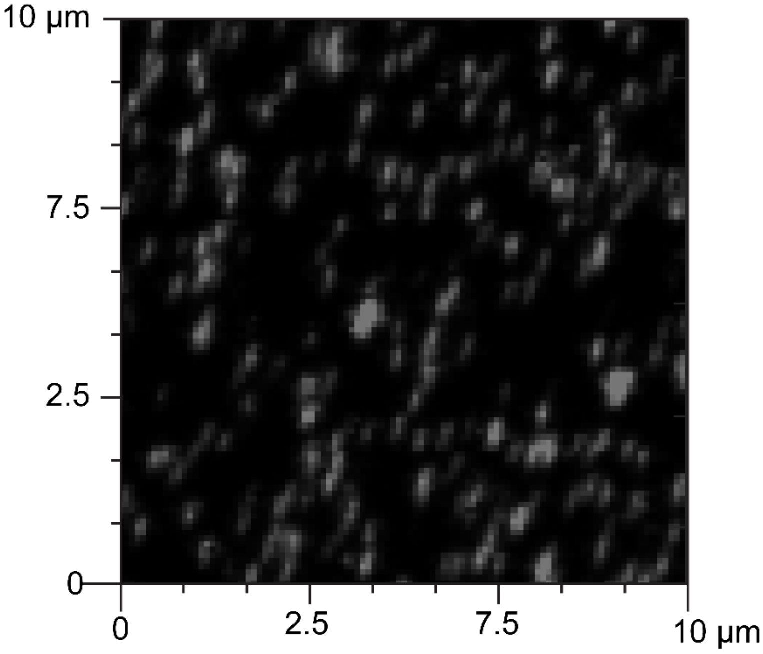

Supplementary Figure 6. *Fluorescence image of PPEB-2 chains, showing single-chain density.*

We then anneal this film under varying conditions, which swells it, allowing the chains to become mobile and aggregate. We then record a fluorescence image again and count the number of spots, which gives us the number of aggregates as shown in Supplementary Figure 7.

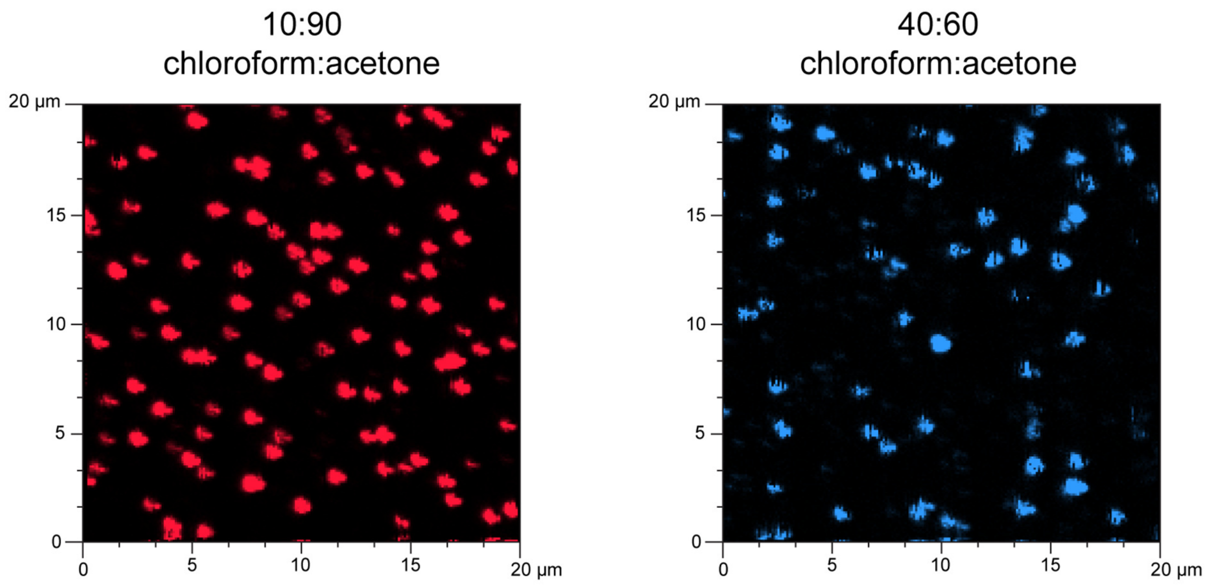

Supplementary Figure 7. *Fluorescence image of PPEB-2 aggregates after solvent vapour annealing, with chloroform:acetone percentages as denoted. Counting of the number of aggregates across multiple images allows the number of chains per aggregate to be deduced.*

Simple division allows us to ascertain than on average each aggregate thus contains a certain number of chains. We repeat this across several images and under the different SVA conditions to allow us to determine the number of chains per aggregate, with results as shown below in Supplementary Table 3.

Supplementary Table 3: Average number of PPEB-2 chains per aggregate for two SVA conditions.

SVA conditions (chloroform:acetone ratio)	Average number of chains per aggregate	Standard deviation
10:90	8.83	0.32
40:60	6.44	0.19

Knowing the average number of polymer chains contained in an aggregate (54 for PPEB-1[6] and the values as denoted above for PPEB-2), the average molecular weight of a chain (40 kDa with PDI of 1.45 for PPEB-1 and 66 kDa with PDI 1.05 for PPEB-2)[7, 8] and assuming[3] a mass density of 1 g cm^{-3} we can then deduce the average volume of an aggregate. In our case, we get 3.59×10^{-18} cm^3 for 54 chains of PPEB-1, 9.67×10^{-19} cm^3 for 9 chains and 7.06×10^{-19} cm^3 for 6 chains of PPEB-2.

7. PPEB aggregate traces

Supplementary Figure 8 plots PL intensity traces for PPEB-1 and PPEB-2, with 10 ms binning. Traces do not show bleaching and the aggregate emission is relatively stable.

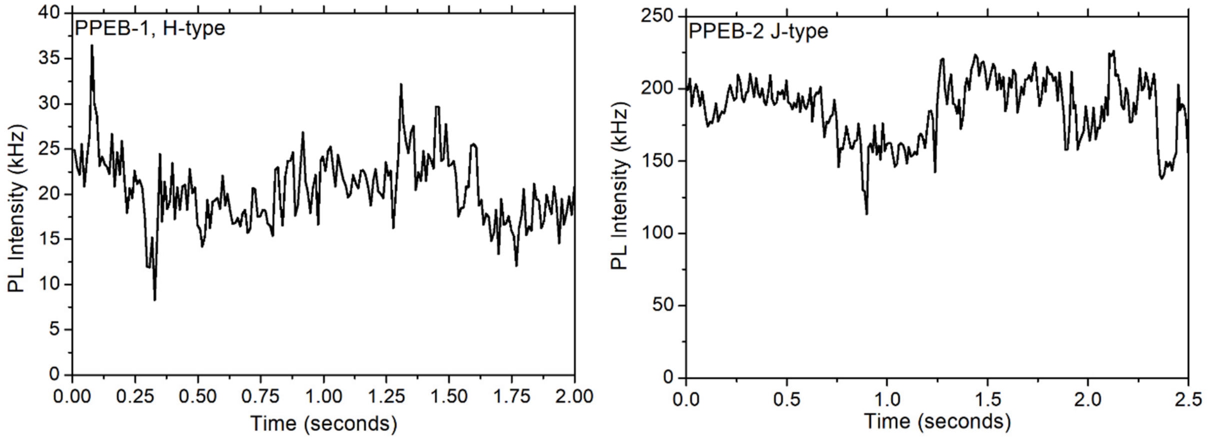

Supplementary Figure 8. *PL intensity traces for PPEB-1 (left) and PPEB-2 (right) aggregates. The data is binned at 10 ms time resolution.*

8. Independence of psTRAB of PPEB aggregates on excitation density

Shown in Supplementary Figure 9 are accumulated psTRAB decays for PPEB-1 H-type aggregates each at two values of excitation fluence: 1.78 μJ cm^{-2} (631 aggregates measured) and 3.56 μJ cm^{-2} (90 aggregates measured). The fluence is determined by measuring the power at the objective, using a visible-range silicon photodiode detector on a Coherent Fieldmaster II power meter, and estimating the diffraction-limited spot size with the 1.35 NA objective as ~366 nm diameter. We find that at these two fluences, the psTRAB results are essentially the identical. Obviously, the range over which such a fluence dependence can be performed is rather limited since the photon correlation spectroscopy requires substantial fluence to begin with, and higher fluences accelerate photodegradation.

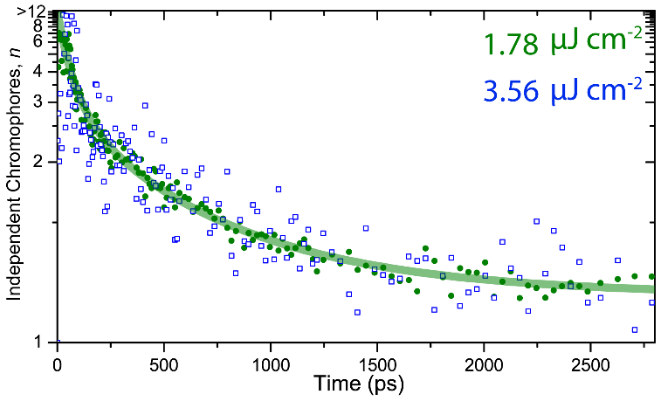

Supplementary Figure 9. *Power dependence of the psTRAB results for 631 and 90 single PPEB-1 H-type aggregates, showing identical data for fluences of 1.78 μJ cm^{-2} (solid green circles) and 3.56 μJ cm^{-2} (open blue squares) along with the guide-for-the-eye (green line).*

9. Evolution with time of photon-antibunching histograms

Shown in Supplementary Figure 10 are the extracted photon-antibunching histograms for the J-type (panel b) and H-type (panel c) PPEB aggregates, along with the psTRAB decays as discussed in the main text (panel a, cf. Figure 4b). These five histograms each in panels b,c are extracted for the five time ranges indicated by green shading (0-67 ps, 117-317 ps, 467-667 ps, 817-1417 ps, 2-2.75 ns).

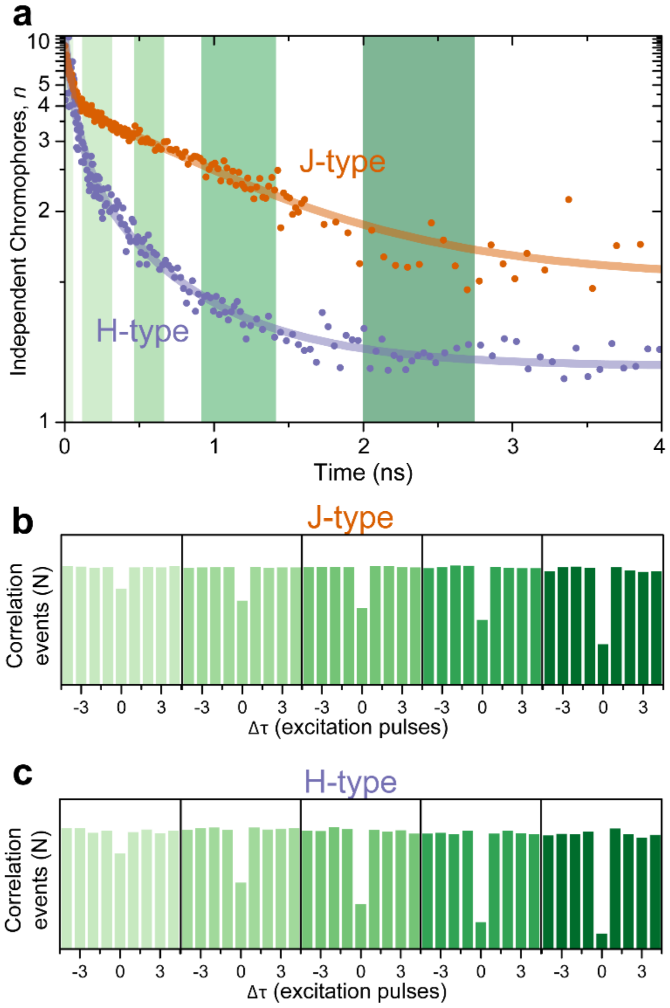

Supplementary Figure 10. *The psTRAB dynamics of the J- and H-type aggregates, discussed in Figure 4b in the main text (a) along with the extracted photon-antibunching histograms for the J-type (panel b) and H-type (panel c) aggregates. The histograms are extracted from the coloured time windows as indicated, going from left to right of: 0-67 ps, 117-317 ps, 467-667 ps, 817-1417 ps, and 2-2.75 ns.*

10. Quantum-statistical description of psTRAB photon correlations

Definition of the correlation-ratio observable

For n_{dyes} chromophores emitting into free space without a cavity, the second-order correlation of emitted photons detected at times t and $t+\Delta t$ can be calculated from the second-order correlation of chromophore deexcitation

$$G^{(2)}(t,t+\Delta t)=\sum_{i,j=0}^{n_{\text{dyes}}}\langle a_i^\dagger(t)a_j^\dagger(t+\Delta t)a_j(t+\Delta t)a_i(t)\rangle,$$

where $a_i(t)$ is the operator on the $2^{n_{\text{dyes}}}$ dimensional Hilbert space that destroys the excitation of chromophore i, each chromophore being modeled as a two-level system with only ground and excited states. For the case of excitation by laser impulses at regularly spaced times $t_0,\ t_0\pm T, t_0\pm 2T, \ldots$ we define the *central-bin* and the *lateral-bin delay-integrated second-order correlation* at time $t_0<t<t_0+T$ as

$$G_{\text{c}}^{(2)}(t)=\int_0^{T-t}G^{(2)}(t,t+\Delta t)d(\Delta t) \quad \text{and}$$

$$G_{\text{l}}^{(2)}(t)=\int_T^{2T-t}G^{(2)}(t,t+\Delta t)d(\Delta t),$$

respectively. Finally, the *central-to-lateral-bin second-order correlation ratio* is defined as $G_{\text{c}}^{(2)}(t)/G_{\text{l}}^{(2)}(t)\equiv N_{\text{c}}(t)/N_{\text{l}}(t)$.

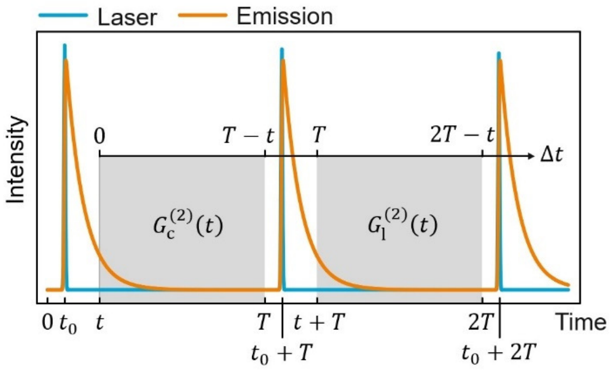

Supplementary Figure 11. *Visualization of the temporal integration ranges used for $G_{\text{c}}^{(2)}(t)$ and $G_{\text{l}}^{(2)}(t)$. The first photon is detected at time $t>t_0$, and the second photon is detected at time $t+\Delta t$ chosen from either the same central laser repetition period (left gray domain) or the lateral laser repetition period (right gray domain).*

Quantum-mechanical description of n_{dyes} coupled chromophores under optical driving

We describe each chromophore by a two-level Hilbert space $\mathcal{H}_2$ with basis states $|0\rangle$ (ground state) and $|1\rangle$ (singlet excited state) and use the usual definitions for the lowering operator $a=|0\rangle\langle 1|$ and the raising operator $a^\dagger=|1\rangle\langle 0|$. The Hamiltonian for a single dye chromophore coupled to a short-impulse laser light field $\mathcal{E}(t)=\mathcal{E}_0\sin(\omega t)\sum_{j\in\mathbb{Z}}e^{-2\ln(2)\left(\frac{t+j\cdot T}{\Delta t_{\text{fwhm}}}\right)^2}$ is

$$H=\hbar\omega_0\, a^\dagger a+\hbar\Omega(t)\left(a+a^\dagger\right)\sin(\omega t),$$

where $\hbar\omega_0$ is the excited-state energy, $\Omega(t) = \frac{\mu}{\hbar}\mathcal{E}_0 \sum_{j\in\mathbb{Z}} e^{-2\ln(2)\left(\frac{t+jT}{\Delta t_{\mathrm{fwhm}}}\right)^2}$ is the slowly-varying Rabi frequency and μ is the transition dipole strength. Using the Hermitian operator $A = \omega\, a^\dagger a$ and the corresponding unitary transformation

$$U = e^{iAt} = e^{i\omega t} a^\dagger a + a\, a^\dagger,$$

the Hamiltonian can be transformed as

$$\widetilde{H} = UHU^\dagger + i\hbar \frac{dU}{dt} U^\dagger = UHU^\dagger - \hbar A,$$

resulting in the rotating-frame Hamiltonian

$$\widetilde{H} = \hbar(\omega_0 - \omega) a^\dagger a + \frac{\hbar\Omega(t)}{2i}\left(a\left(1 - e^{-2i\omega t}\right) - a^\dagger(1 - e^{2i\omega t})\right).$$

In the rotating-frame approximation and for resonant excitation $\omega = \omega_0$, high-frequency components at $\pm 2\omega$ are dropped and the Hamiltonian reduces to

$$\widetilde{H} = \frac{\hbar\Omega(t)}{2i}\left(a - a^\dagger\right).$$

Generalizing for the case of n_{dyes} chromophores, the Hilbert space is constructed as the tensor product $\mathcal{H}_2 \otimes \ldots \otimes \mathcal{H}_2$ and

$$\widetilde{H} = \frac{\hbar\Omega(t)}{2i} \sum_i \left(a_i - a_i^\dagger\right).$$

Since we are not interested in the coherences, calculation of the system dynamics in the rotating frame is sufficient to derive expectation values for the relevant photon correlation. In order to account for spontaneous emission and singlet-singlet annihilation, we describe the system state by a $2^{n_{\mathrm{dyes}}} \times 2^{n_{\mathrm{dyes}}}$ dimensional density matrix $\rho(t)$ and treat its time dynamics on the level of a Lindblad master equation[9]

$$\frac{d}{dt}\rho(t) = \mathcal{L}(t)\rho(t) = -\frac{i}{\hbar}[H(t), \rho(t)] + \sum_{j=1}^{n_{\mathrm{dyes}}} \frac{1}{2}\left(2c_j\rho(t)c_j^\dagger - \rho(t)c_j^\dagger c_j - c_j^\dagger c_j \rho(t)\right) + \sum_{j\neq k} \frac{1}{2}\left(2d_{jk}\rho(t)d_{jk}^\dagger - \rho(t)d_{jk}^\dagger d_{jk} - d_{jk}^\dagger d_{jk}\rho(t)\right).$$

The quantum-jump operators for spontaneous emission are $c_j = \sqrt{k_{\mathrm{r}}}a_j$ and those for singlet-singlet annihilation are $d_{jk} = \sqrt{k_{\mathrm{ET}}}a_j a_k^\dagger a_k$. In the context of our constrained two-level description, the latter operator effectively models energy transfer from chromophore j to chromophore k at a rate $k_{\mathrm{ET}} = \frac{1}{2}k_{\mathrm{SSA}}$, assuming that after excursions to higher excited states (not included in our Hilbert space), the receiving chromophore immediately returns to the first excited state. Here, k_{SSA} is the total exponential decay rate at which an individual pair of excitations decays by singlet-singlet annihilation to a single excitation.

Two-time correlations for the excitations (i.e. for the emitted photons) can be calculated in the context of the validity of the Lindblad equation by using the quantum-regression theorem[10]

$$\langle a_i^\dagger(t) a_j^\dagger(t+\tau) a_j(t+\tau) a_i(t)\rangle = \mathrm{Tr}\left(a_j^\dagger a_j \Lambda_i(t, t+\tau)\right),$$

where $\Lambda_i(t, t+\tau)$ describes the system that at time t is reduced by one excitation on chromophore i and then follows the same differential equation as the original density matrix.

$$\Lambda_i(t,t) = a_i\rho(t)a_i^\dagger$$

$$\frac{\partial}{\partial\tau}\Lambda_i(t,t+\tau) = \mathcal{L}(t+\tau)\Lambda_i(t,t+\tau).$$

The two-time correlation can thus be calculated by the product of probabilities of removing one excitation from the system at time t, and removing the other one at time $t+\tau$ from the system that was disturbed at time t by reducing the number of excitations by one.

As an example, the system dynamics is solved by numerical integration for the case of three chromophores on the *Mathematica* computer algebra platform (Wolfram Research, USA). Parameters where chosen to model chromophores with unity quantum efficiency, a radiative rate of $k_\mathrm{r} = 0.25 \cdot 10^9\ \mathrm{s}^{-1}$, and a singlet-singlet annihilation rate of $k_\mathrm{SSA} = 0.25 \cdot 10^9\ \mathrm{s}^{-1}$. Excitation conditions were $\Delta t_\mathrm{fwhm} = 50$ ps laser impulse width, $T = 50$ ns repetition interval and an instantaneous peak Rabi frequency of $2 \cdot 10^9\ \mathrm{s}^{-1}$. The number of excitations in the coupled set of chromophores can be calculated as $\mathrm{Tr}(\rho(t)\sum_i a_i^\dagger a_i)$.

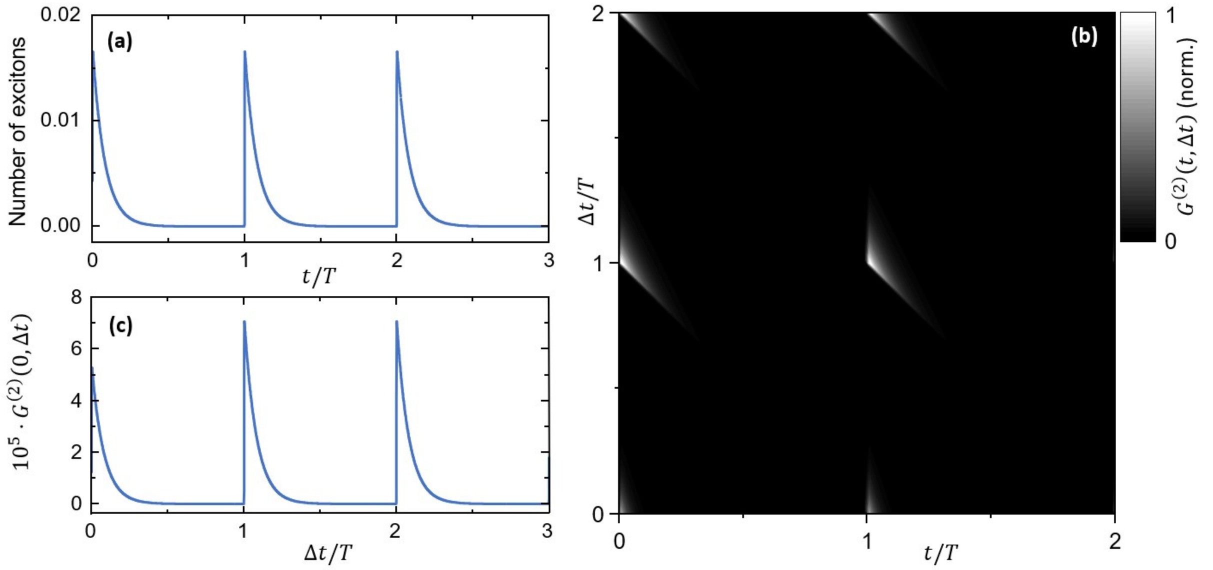

Supplementary Figure 12. *(a) Number of excitons in a three-chromophore system as a function of time t under pulsed laser excitation. Due to the low excitation strength, singlet-singlet annihilation has no measurable impact on the emission intensity. (b) Normalized two-time correlation $G^{(2)}(t, t+\Delta t)$ as a function of t and Δt in units of the pulse repetition period T. (c) For delay times $\tau < t - T$, the correlation is reduced due to the loss of one exciton in the system at time t. The example shows the case of $t = 0$.*

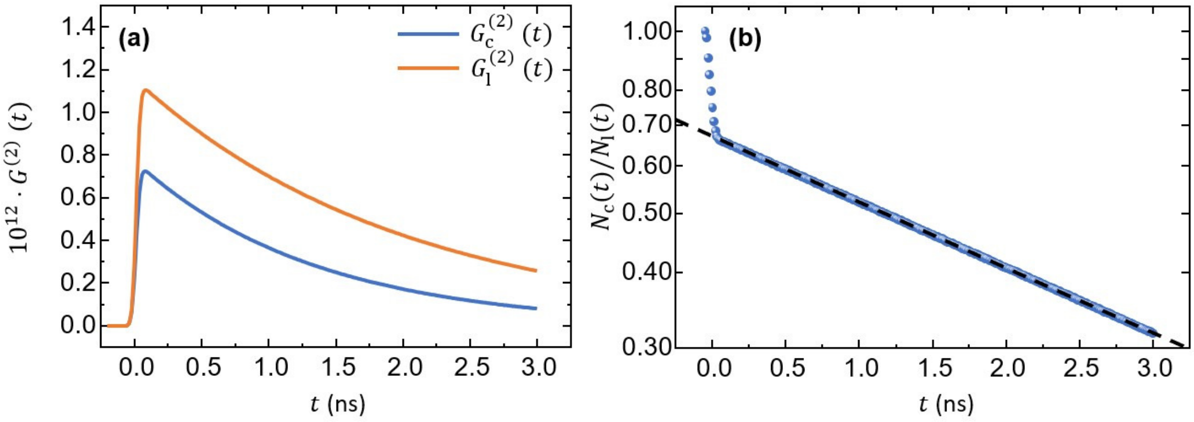

Supplementary Figure 13. *(a) Central-bin photon correlation* $G_c^{(2)}(t)$ *(blue) and lateral-bin photon correlation* $G_l^{(2)}(t)$ *(orange) as a function of time* t *after laser excitation. (b) After the end of the excitation pulse, the correlation ratio* $N_c(t)/N_l(t) = G_c^{(2)}(t)/G_l^{(2)}(t)$ *(blue) follows the exponential decay* $\frac{n_{dyes}-1}{n_{dyes}} e^{-k_{SSA}t}$ *that directly reflects the annihilation dynamics of exciton pairs (dashed line). The initial decay of the correlation ratio from 1 to* $(n_{dyes} - 1)/n_{dyes}$ *is due to the high probability of re-excitation if the first photon is detected early during the excitation pulse.*

Analytical treatment

The system can be treated analytically for the case of negligibly short excitation impulses, assuming that the dynamics is treated in the limit of vanishing Rabi frequency $\Omega \to 0$. We first discuss the the $n_{dyes} = 2$ system analytically, assuming that at $t < 0$ the system reaches equilibrium due to optical pumping. In this case, the total number of excitations in the system is $p_0 n_{dyes}$, where the probability p_0 for a single dye chromophore to be excited depends on the excitation intensity. From the probability of detecting a photon we find the emission intensity $I(t) = k_r \mathrm{Tr}\left((a_1^\dagger a_1 + a_2^\dagger a_2)\rho(t)\right)$ to be

$$I(t) = \frac{k_r}{2} e^{-(2k_r + k_{SSA})t} p_0 n_{dyes} \left(\frac{k_{SSA} p_0 n_{dyes}}{4(k_r + k_{SSA})} + \left(1 - \frac{k_{SSA} p_0 n_{dyes}}{4(k_r + k_{SSA})}\right) e^{(k_r + k_{SSA})t}\right)$$

Experimentally, one works in the low-excitation regime $p_0 n_{dyes} \ll 1$, and more specifically $k_{SSA} p_0 n_{dyes} \ll 4(k_r + k_{SSA})$. In this limit, the intensity is $I(t) \approx k_r p_0 e^{-k_r t}$. Since in this case the system is only ever singly excited, the photon correlations are calculated as

$$G_c^{(2)}(t, t+\tau) = k_r^2 n_{dyes}(n_{dyes} - 1) \mathrm{Tr}\left(a_2^\dagger a_2 \, \Lambda(t, t+\tau)\right) \cdot \mathrm{Tr}(a_1^\dagger a_1 \rho(t))$$

$$G_l^{(2)}(t, t+\tau) = k_r^2 n_{dyes}^2 \mathrm{Tr}\left(a_2^\dagger a_2 \, \rho(t+\tau)\right) \cdot \mathrm{Tr}\left(a_1^\dagger a_1 \rho(t)\right).$$

Since the analytical description lacks the action of the pulsed excitation, the central-bin and lateral-bin correlations must be calculated differently than in the experimental case. For the central-bin correlation, annihilation of one excitation at time t on chromophore 1 defines the new initial state $\Lambda(t, t) = a_1 \rho(t) a_1^\dagger$. There are $n_{dyes} = 2$ choices for the first emitting chromophore and only $(n_{dyes} - 1) = 1$ choice for the second, since the first chromophore cannot be reexcited. For the lateral-bin correlation, annihilation of the first exciton on one of the n chromophores does not change the density matrix, since the system fully recovers from one to the next excitation pulse. The number of available chromophores to emit the second photon is thus also $n_{dyes} = 2$. In the limit of $p_0 n_{dyes} \ll 1$, one finds

$$G_c^{(2)}(t, t+\tau) = \frac{k_r^2}{4} n_{dyes}(n_{dyes} - 1)\, p_0^2 e^{-(k_r+k_{ET})t} e^{-(k_r+k_{ET})t} e^{-k_r\tau}$$

$$G_l^{(2)}(t, t+\tau) = \frac{k_r^2}{4} n_{dyes}^2 p_0^2 e^{-k_r t} e^{-k_r t} e^{-k_r\tau}$$

Integration over the delay time τ then yields

$$G_c^{(2)}(t) = \int_0^\infty G_c^{(2)}(t, t+\tau) d\tau = \frac{k_r}{4} n_{dyes}(n_{dyes} - 1)\, p_0^2 e^{-(2k_r+k_{SSA})t}$$

$$G_l^{(2)}(t) = \int_0^\infty G_l^{(2)}(t, t+\tau) d\tau = \frac{k_r}{4} n_{dyes}^2 p_0^2 e^{-2k_r t}$$

and a correlation ratio of

$$\frac{N_c(t)}{N_l(t)} = \frac{G_c^{(2)}(t)}{G_l^{(2)}(t)} = \frac{n_{dyes}-1}{n_{dyes}} e^{-k_{SSA}t}.$$

These results for $n_{dyes} = 2$ can be directly rationalized for higher numbers of chromophores and the case of non-radiative decay at rate k_{nr}. Under conditions of $p_0 n_{dyes} \ll 1$, the probability to find two excited chromophores after each of two adjacent excitation pulses is negligible, even for systems with $n_{dyes} > 2$ chromophores. Thus, lateral-bin correlations derive from chromophore ensembles with only *one* excitation present and are devoid of the effect of singlet-singlet annihilation. On the other hand, central-bin correlations can only exist if indeed two or more excitations are present after the laser excitation pulse. Since $p_0 n_{dyes}$ is small, cases with more than two excitations need not be considered. Obviously, the probability to find two excitations decays proportionally to $e^{-k_{SSA}t}$, where k_{SSA} is the *exponential decay rate for pairs of singlet excitons*, such that in a macroscopic ensemble with a high number of excitons n_{exc}, one would expect a non-exponential decay according to $\frac{d}{dt} n_{exc} = -n_{exc}(n_{exc} - 1)k_{SSA}$. Microscopically however, each excited chromophore is exponentially quenched by each other excited chromophore at the *energy transfer rate* $k_{ET} = k_{SSA}/2$. Applying the quantum-regression theorem, the probability of finding the second photon emission at time $t + \tau$ for the central-bin second-order correlation is calculated by assuming that the number of excitons is *reduced to one* at time t. There is a total number of n chromophores to choose from as source of the first detected photon, and neglecting the possibility of double-excitations of individual chromophores, a total of $(n_{dyes} - 1)$ chromophores exist that can potentially carry the second exciton. The central-bin second-order correlation is thus calculated as

$$G_c^{(2)}(t, t+\tau) = n_{dyes} k_r\, p_0\, e^{-(k_r+k_{nr}+k_{ET})t} \cdot k_r(n_{dyes} - 1) p_0 e^{-(k_r+k_{nr}+k_{ET})t} e^{-(k_r+k_{nr})\tau}$$

$$G_c^{(2)}(t) = n_{dyes}(n_{dyes} - 1) p_0^2 k_r^2 \tau_{PL} e^{-2(k_r+k_{nr})t} e^{-k_{SSA}t},$$

where $\tau_{PL} = 1/(k_r + k_{nr})$ is the single-chromophore photoluminescence lifetime. For the lateral-bin correlation, the only relevant contribution comes from the configuration where there is exactly one exciton in the chromophore system when the first photon is emitted and exactly one photon in the chromophore system when the second photon is emitted after the next (lateral) excitation pulse. Thus, singlet-singlet quenching does not contribute to the lateral-bin second-order correlation. Again, n_{dyes} chromophores can potentially emit the first photon, but in contrast to the calculation of the central-bin correlation, for the lateral-bin correlation the system is re-excited in-between the two photon detection events, and thus the number of chromophores that can emit the second photon is also n_{dyes}:

$$G_{\mathrm{l}}^{(2)}(t,t+\tau) = n_{\mathrm{dyes}} k_{\mathrm{r}}\, p_0\, e^{-(k_{\mathrm{r}}+k_{\mathrm{nr}})t} \cdot k_{\mathrm{r}} n_{\mathrm{dyes}}\, p_0 e^{-(k_{\mathrm{r}}+k_{\mathrm{nr}})t} e^{-(k_{\mathrm{r}}+k_{\mathrm{nr}})\tau}$$

$$G_{\mathrm{l}}^{(2)}(t) = n_{\mathrm{dyes}}^2 p_0^2 k_{\mathrm{r}}^2 \tau_{\mathrm{PL}} e^{-2(k_{\mathrm{r}}+k_{\mathrm{nr}})t}.$$

The resulting central-to-lateral-bin second-order correlation ratio for the general case of n_{dyes} chromophores is thus again simply

$$\frac{N_{\mathrm{c}}(t)}{N_{\mathrm{l}}(t)} = \frac{G_{\mathrm{c}}^{(2)}(t)}{G_{\mathrm{l}}^{(2)}(t)} = \frac{n_{\mathrm{dyes}}-1}{n_{\mathrm{dyes}}} e^{-k_{\mathrm{SSA}}t}.$$

An important observation in this treatment is that the choice of the singlet-singlet annihilation mechanism and the associated decay law $e^{-k_{\mathrm{SSA}}t}$ is entirely arbitrary. The immediate conclusion is that the correlation ratio can be used to directly determine the actual decay function for the doubly-excited system.

Supplementary Table 4: Sequences for the modified DNA origami structure.

5‘ position	Sequence	Comment
0[286]	AAAACGAAAGAGGCTCATTATAC	
11[105]	ACACAACATACGAGGGATGTGGCTATTAATCGGCC	
9[567]	ATCATTTACATAAAAGTATCAAAATTATAAGAAACTTCAATA	
7[567]	CAGCTTTGAATACCAAGTTACAA	
5[455]	CATGCCAGTGAGCGCTAATATCCAATAATAAGAGC	
2[223]	CCGAACTTTAATAAAAGCAAAGCGGATT	
5[497]	TTGAGAATATCTTTCCTTATCACTCATCGAGAACA	
9[315]	CAGATATAGGCTTGAACAGACGTTAGTAAAGCCCAAAAATTT	
5[287]	GCGCAGCGACCAGCGATTATATATCATCGCCTGAT	
8[69]	TCGGTCATACCGGGGGTTTCTGC	
11[219]	GTGCCTGCTTTAAACAGGGAGAGAGTTTCAAAGCGAACCA	
10[457]	AAAAGATAGGGTTGAGTGT	
2[643]	GATAGTGCAACATGATATTTTTGAATGG	
0[347]	AGCGTATCATTCCACAGACCCGCCACAGTTGCAGCAAGCG	
9[483]	ATAATGAATCCTGAGATTACGAGCATGTGACAAAAACTTATT	
8[573]	AAATGCGTTATACAAATTCTTAC	
2[433]	AGGGACAAAATCTTCCAGCGCCAAAGAC	
7[63]	GCCCGCACAGGCGGCCTTTAGTG	
4[377]	CTATTTCGGAACGAGTGAGAATA	
0[698]	TTTTTCGGGAGCTAAACAGGTTGTTAGAATCAGAGTTTTT	
4[587]	CATCGGGAGAAATTCAAATATAT	
7[506]	AAATCAGCCAGTAATAACACTATTTTTGAAGCCTTAAATC	
7[170]	TTTTTATCCAATAAATCTCTACCCCGGTAAAACTAGCATG	
5[161]	GTATACAGGTAATGTGTAGGTAGTCAAATCACCAT	
4[396]	AACAGAGTGCCTGGGGTTTTGCTCACAGAAGGATTAGGAT	
3[350]	GTCACCAGTACAAGGTTGAGGCA	
5[581]	ACATCATTTAAATTGCGTAGAAACAGTACCTTTTA	
5[623]	ATACCCTTCGTGCCACGCTGAACCTTGCTGAACCT	
8[130]	GGGCGTGAAATATTAGCGCCATTCGC	
9[357]	TCTTATACTCAGAAAGGCTTTTGATGATATTGACACGCTATT	

11[345]	GAGAGCCTCAGAACCGCATTTTCTGTAACGATCTAAAGTT	
5[329]	TTCATTTTCTGCTAAACAACTGAACAACTAAAGGA	
8[489]	AAAACGGAATACCCAAAAGAACT	
0[202]	GACCGGAAGCAATTGCGGGAGAA	
3[182]	GCTAAATCGGTTTGACTATTATA	
3[392]	ATATTCACAAACAAATTCATATG	
6[69]	AAAAGTGTCAGCAACAATTGCAGGCGCT	
11[567]	ACCATCACCCAAATAAACAGTTCATTTGATTCGCC	
7[590]	AATCGTTGAGTAACATTGGAATTACCTAATTACATTTAAC	
11[93]	GCTCAAGTTGGGTAACGGGCGGAAAAATTTGTGAGAGATA	
0[305]	ACTACCTTTAAACGGGTAACAGGGAGACGGGCA	
4[270]	TCAACATCAGTTAAATAGCGAGAGTGAGACGACGATAAAA	
6[153]	TAAATCGGTTGGTGCACATCAAAAATAA	
10[163]	TCAGCTAACTCACATTAAT	
7[231]	TGCAACACTATCATAACCCTCGT	
4[438]	ACCAAATTACCAGGTCATAGCCCCGAGTTTCATCGGCAT	
8[195]	TTAACAAGAGAATCGATGAACGG	
3[625]	AGACAACCTGAACAGTATTCGAC	
8[363]	TGAACAGCTTGATACCGATAGTT	
0[412]	TCACCGTCACCGGCGCAGTCTCT	
4[706]	TTTTTGTCCATCACGCAAATTCCGAGTAAAAGAGTCTTTTTT	
11[315]	ACAGCTGATTGCCCGTCGCTGCGCCCACACGTTGA	
8[424]	CGGAAGCACGCAAACTTATTAGCGTT	
0[431]	ATTCAAGGGGAAGGTAAATGTGGCAAATAAATC	
3[602]	TGATTATCAGATATACGTGGCAC	
4[545]	TGACCTAAATTTTTAAACCAAGT	
3[679]	GGTTGCTTTGACGAGCACGTTTTT	
6[573]	TGATTTAGAAAACTCAAGAGTCAATAGT	
11[441]	AAAAGAATAGCCCGATACATACGCAGTAAGCTATC	
8[634]	TACATAAATTCTGGGCACTAACAACT	
3[541]	CATAGTTAATTTGTAAATGTCGC	
11[147]	TGCCTAATGAGTGAGAAAAGCTCATATGTAGCTGA	
9[651]	AATAGCTGTCACACGCAACGGTACGCCAGCGCTTAATGTAGTA	
0[557]	TACCTAATATCAAAATCATTCAATATTACGTGA	
4[60]	TCAGAGGTGTGTCGGCCAGAATGAGTGCACTCTGTGGT	
3[476]	TTTTTTGTTTAATAAAGTAATTC	
8[382]	AAGTAAGAGCCGCCAGTACCAGGCGG	
3[79]	GTGGAACGACGGGCTCTCAACTT	
4[102]	CCAGCCAAACTTCTGATTGCCGTTTTGGGTAAAGTTAAAC	
3[121]	AATCAGTTAAAACGTGGGAGAAA	
3[224]	GCATCAAAAAGAAGTAAATTGGG	
7[212]	TTTCACGAGAATGACCATTTTCATTTGGTCAATAACCTGT	
8[678]	CCTACATACGTAGCGGCCAGCCATTGCAACAGGTTTTT	
5[539]	TTCGCTATTCGCAAGACAAAGTTAATTTCATCTTC	
7[17]	TTTTTATCCAGCGCAGTGTCACTGC	

8[298]	CATAGAATTTGCGGTTTGAAAGAGGA	
10[79]	GTATGTGAAATTGTTATCC	
7[273]	ACTACTTAGCCGGAACGAGGCGC	
11[387]	GGCGACACCACCCTCAGGTTGTACTGTACCGTTCCAGTAA	
6[447]	TTACCTCTTAGCAAATTTCAACCGATTG	
8[508]	GGTTTGCGCATTTTAACGCGAGGCGT	
10[415]	CCTCCGAAATCGGCAAAAT	
4[480]	TAAGCCAGAGAGCCAGAAGGAAACTCGATAGCCGAACAAA	
0[179]	GCCTTATACCCTGTAATACCAATTCTTGCGCTC	
9[147]	CATTCAACCCAAAATGTAGAACCCTCATGAATTAGTACAACC	
7[525]	TATGTGATAAATAAGGCGTTAAA	
0[454]	AGACGGGAGAATTGACGGAAATT	
11[681]	AAAGGGCGCTGGCAAGTATTGGC	
4[228]	GAGCTTAAGAGGTCCCAATTCTGCAATTCCATATAACAGT	
3[331]	TACCGGGATAGCAATGAATATAT	
4[335]	ATTGCGAATAATGTACAACGGAG	
2[265]	TATGCATTACAGAGGATGGTTTAATTTC	
4[564]	TTTAGAACGCGAATTACTAGAAAACTATAAACACCGGAAT	
11[597]	GAGGTAACGTTATTAATTTTAAAACAAATAATGGAAGGGT	
5[25]	TTTTTCCGGTGCAGCACCGATCCCTTACACTTGCC	
1[17]	TTTTTTGGTAATGGGTAACCATCCCACTTTTT	
8[531]	AACGAACCTCCCGACTTGCGGGA	
0[515]	CTGAAAACCTGTTTATCAAACATGTAACGTCAA	
8[592]	AAAATTTTTTAAAATGAGCAAAAGAA	
7[609]	ATTTGGCAAATCAACAGTTGAAA	
11[639]	CCGATAATAAAAGGGACTTAACACCGCGAACCACCAGCAG	
3[583]	GGAATCGGAACATTGCACGTTAA	
2[349]	TGTAGGGGATTTAGTAACACTGAGTTTC	
3[434]	AAAAGGGCGACAATTATTTATCC	
5[371]	ATCAGAGCCTTTAACGGGGTCTTAATGCCCCCTGC	
7[338]	GGAGCAGCCACCACCCTTCGCATAACGACAATGACAACAA	
3[56]	ATCAGCGGGGTCAGCTTTCAGAG	
0[473]	AAAAAAGGCAGCCTTTACAATCTTACCAGTTTG	
6[698]	TTTTTAACAATATTACCGTCGCTGGTAATATCCAGTTTTT	
8[88]	AGCCTCCCCAGGGTCCGGCAAACGCG	
6[405]	CAAGTGCTGAGTAAGAAAATAAATCCTC	
7[632]	GGAATAACAGAGATAGACATACAAACTTGAGGATTTAGAA	
0[76]	GACTTTCTCCGTGGCGCGGTTG	
2[97]	GCGAAAGACGCAAAGCCGCCACGGGAAC	
4[648]	GCATCGAGCCAGATATCTTTAGGACCTGAGGAAGGTTATC	
4[606]	ACAGTTTTTCAGATTTCAATTACCGTCGCAGAGGCGAATT	
7[548]	TAAGATCTGTAAATCGTTGTTAATTGTAAAGCCAACGCTC	
11[555]	CCCACATGTGAGTGAATAACTGATGCTTTTAACCTCCGGC	
9[399]	ATAAGAAGCCACCCAAACTTGAGCCATTATCAATACATCAGT	
11[189]	ACTGCCCGCTTTCCTGAAAAGCTATATTTTAAATA	

3[499]	TGTCCAAGTACCAGAAACCCCAG	
4[209]	AATGCTGTAGCTGAGAAAGGCCG	
7[357]	GTGTATTAAGAGGCTGAGACTCC	
8[237]	GCTTGACCATTAGATACATTTCG	
9[609]	GATGAATAAATCCTGTAGGTGAGGCGGTAGCGTAAGTCCTCA	
0[328]	TTGTCGTCTTTCTACGTAATGCC	
11[513]	CTCCAATTTAGGCAGAGACAATCAATCAAGAAAAATAATA	
3[560]	AAGACGCTGAGACCAGAAGGAGC	
7[42]	GCGCCTGAATGCCAACGGCCCAGCCTCCCGCGTGCCTGTTCTTCTTTTT	
0[370]	GCGTCATACATGCCCTCATAGTT	
11[303]	GTGAGTTAAAGGCCGCTGACACTCATGAAGGCACCAACCT	
3[373]	GGTCACGCCAGCACAGGAGTTAG	
4[51]	GGGTTACCTGCAGCCAGCGGTGTTTTT	
7[254]	TTACCAATAAGGCTTGCAGTGCGGAAGTTTAGACTGGATA	
8[466]	GGCATAAGCGTCTTCGAGGAAACGCA	
3[247]	CTTGAAAACACCCTAACGGCATA	
8[405]	GGTGCCGTCGAGAGGGTTGATAT	
10[331]	TCGTTCACCGCCTGGCCCT	
8[615]	GTTGAAACAAACATCAAGAAAAC	
6[531]	GACCGTCGAACGGGGAAGCTAATGCAGA	
6[363]	TGAAATTGTTTCAGGGAACTACAACGCC	
10[625]	AACACCCTAAAGGGAGCCC	
6[279]	CATGTCAGAGATTTGATGTGAATTACCT	
11[429]	CCCTTCATATAAAAGAACGTAGAGCCTTAAAGGTGAATTA	
11[651]	TTGACGGGGAAAGCTTCACCAGAAATGGCATCACT	
6[615]	GTCAGTCGTTTAACGAGATGGCAATTCA	
7[422]	AGCGCCACCACGGAATACGCCTCAGACCAGAGCCACCACC	
4[312]	ATTTGCCAAGCGGAACTGACCAACGAGTCAATCATAAGGG	
8[550]	CAGTAAGAACCTTGAGCCTGTTTAGT	
4[503]	AGCAAGCCGTTTAAGAATTGAGT	
2[601]	TCAATAATAAAGTGTATCATCATATTCC	
9[21]	TTTTTGCGTCCGTGCCTGCATCAGACGTTTTT	
11[483]	GAACAAGAGTCCACCAATTTTTTAGTTGTCGTAGG	
10[499]	CTATATTAAAGAACGTGGA	
4[186]	GAGACAAAGATTATCAGGTCATTGACGAGAGATCTACAAA	
9[63]	TTCACCTAGCGTGGCGGGTGAAGGGATACCAGTGCATAAAAA	
11[609]	AGCACTAAATCGGATCGTATTTAGACTTATATCTG	
4[293]	AAATTGTGTCGAGAATACCACAT	
3[667]	GGCGCCCCGCCGAATCCTGAGAAGTGAGGCCGATTAAAGG	
3[205]	GTCAGAATCAGGCAGGATTCGCG	
0[622]	AAGATAAAACAGTTGGATTATAC	
6[111]	TCAGGTGAAATTTCTACGGAAACAATCG	
10[205]	AGCAGTCGGGAAACCTGTC	
6[489]	AATCATAATAACCCGGCGTCAAAAATGA	
0[496]	TCCCATCCTAATGAGAATAACAT	

0[221]	CGAGCACAGACTTCAAATACCTCAAAAGCTGCA	
9[231]	TTAGTGTGAATCCCTCTAATAAAACGAAAGAACGATGAATTA	
4[629]	CAAATATCAAACCAGATGAATAT	
0[664]	GATTTTAGACAGGCATTAAAAATA	
10[667]	AGACGGCGAACGTGGCGAG	
0[599]	TTCTGGAATAATCCTGATTTTGCCCGGCCGTAA	
3[23]	TTTGCAACCAGCTTACGGCGGTGGTGAGGTTTCAGTTGAGGATCCTTTTT	
8[340]	GCGCCCGCACCCTCTCGAGGTGAATT	
7[674]	GCCTTACGCTGCGCGTAAAATTATTTTTTGACGCTCAATC	
7[86]	ATGAATCCCAGTCACGATCGAACGTGCCGGCCAGAGCACA	
5[245]	CGCCTGACGGTAGAAAGATTCTAATGCAGATACAT	
8[657]	GTATTAGAGCCGTCAATAGATAA	
3[308]	CTAAAGACTTTTAGGAACCCATG	
2[702]	TTTTTTATAACGTGCTTTCCTCTTTATAACAGTACTAT	
4[671]	TACTTCTTTGATAAAAATCTAAA	
2[391]	ATTAAAATAAGTGCGACGATTGGCCTTG	
9[189]	GAGCAAGGTGGCATTTACTCCAACAGGTTCTTTACGTCAACA	
4[167]	CAATATGATATTGATGGGCGCAT	
7[147]	GCTAATGCCGGAGAGGGTAGCTA	
7[464]	AAGCACAGAGCCTAATTATTGTTAGCGATTAAGACTCCTT	
8[172]	TAATCGTAGCATTACCTGAGAGTCTG	
0[580]	TAGAACCTACCAGTCTGAGAGAC	
4[354]	GAAAGTTCAACAATCAGCTTGCTTAGCTTTAATTGTATCG	
8[46]	CAGCATCAACCGCACGGCGGGCCGTT	
2[181]	TTATGGCCTGAGCACCTCAGAGCATAAA	
3[644]	CTATTAGTCTTTCGCCGCTACAG	
8[111]	CTTTTTTTCGTCTCGTCGCTGGC	
11[231]	TTAATGAATCGGCCATTCATTCCAATACGCATAGT	
3[518]	AACAACATGTTCATCCTTGAAAA	
5[77]	AACGTTGTAGAAACAGCGGATAGTTGGGCGGTTGT	
10[706]	TTTTTAGGAGCGGGCGCTAGGAAGGGAAGAAAGCGAATTTTT	
9[441]	TGCCATACATAAAGATTAACTGAACACCAACAGCCGGAATAG	
7[189]	GGCTAAAGTACGGTGTCTGGAAG	
6[237]	AAGAGATTCATTTTGTTTAAGAGGAAGC	
5[203]	TGTAAATCATGCTCCTTTTGATAATTGCTGAATAT	
7[315]	AATCCAAAAAAAAAGGCTCCAAAA	
10[583]	TGGCAAGTTTTTTGGGGTC	
2[559]	GAATTATCCAATAACGATAGCTTAGATT	
11[364]	GTCCACGCGCCACCTCACCGTTGAAACA	
11[471]	TGTTCCAACGCTAACGAACAAGTCAGCAGGGAAGCGCATT	
4[522]	ACCGCATTCCAACGGTATTCTAAGCGAGATATAGAAGGCT	
7[380]	TCAAGCAGAACCACCACTCACTCAGGTAGCCCGGAATAGG	
8[447]	ATTCTTTTCATAATCAAAATCAC	
6[321]	AAATCCCCGAAACAATTCATGAGGAAGT	
10[541]	CATTCTATCAGGGCGATGG	

10[373]	TACCTGGTTTGCCCCAGCA	
5[413]	AGAGTTTATACCAGTAGCACCTGAAACCATCGATA	
9[105]	GTCCGTCCTGCAAGATCGTCGGATTCTCTTCGCATTGGACGA	
11[63]	ATAGCTGTTTCCTGGAACGTCCATAACGCCGTAAA	
11[177]	TGCGTACTAATAGTAGTTGAAATGCATATTTCAACGCAAG	
8[702]	TTTTTAAAAACGCTCATGGAAATA	
7[441]	TTGAAGCCCTTTTTAAGAAAAGT	
11[525]	AGGGCGAAAAACCGATTTAACGTAGGGCAAATACC	
2[475]	AAATAGGTAATTTACAAATAAGAAACGA	
9[525]	TTTAGCAAACGCCACAATATAACTATATTCCCTTATAAATGG	
7[399]	TATTGCCTTTAGCGTCAGACTGT	
0[389]	GAATTGTAGCCAGAATGGATCAGAGCAAATCCT	
2[307]	TTCCATTGACCCAAAGAGGCTTTGAGGA	
7[651]	TAAGTAGAAGAACTCAAACTATCG	
2[517]	ACGCGTCGGCTGTAAGACGACGACAATA	
7[483]	GTTTACCGCGCCCAATAGCAAGC	
2[55]	TTCGCCATAAACTCTGGAGGTGTCCAGC	
10[48]	GCAGCACTTTGCTCTGAGCCGGGTCACTGTTGCCCTGCGGCTTTTT	
6[657]	TGCCTGAACAGCAAATGAATGCGCGAACT	
3[163]	TAAAGAGGCAAAATATTTTATAA	
0[538]	TTAGGTTGGGTTATAGATAAGTC	
4[419]	GCAGCACCGTAAGTGCCCGTATA	
8[214]	CAAATGGTTCAGAAGAACGAGTAGAT	
3[415]	GTTTATGTCACATGGGAATCCAC	
0[641]	CCGAACCCCCTAAAACATCGACCAGTTTAGAGC	
8[321]	CCGAACGGTGTACAGACCAGGCG	
3[457]	CAATCCAAAATACTGAACAGTAG	
6[195]	TGCAACTCAAAAGGCCGTACCAAAAACA	
0[95]	CCGGAAGACGTACAGCGCCGCGATTACAATTCC	
11[399]	GTTTGATGGTGGTTCAGAACCCCGCCTCACAGAAT	
11[25]	TTTTTCCGGGTACCGAGCTCGAATTCGTAATCTGGTCA	
0[53]	CGGTAGTACTCAATCCGCTGCTGGTCATGGTC	
8[256]	AAAATTCCATTCAGGCTTTTGCAAAAGAAGTCA	
3[266]	AACTTTAATCATGGGTAGCAACGGCTACGACAGCAACTAAAA	
10[247]	AATAACGCGCGGGGAGAGGCGGTT	
0[251]	TGGGAAGAAAAATCTACGTGCGTTTTAATT	
0[263]	CAGTCTTGATTTTAAGAAC	
8[286]	GACCTTCATTTTGCCAGAGGGGGTAATAGT	
7[296]	AGACGTCGTCACCCTCAGACCTGCTC	
4[461]	AAGAAACAATGACCGGAAACGTC	biotin labeled
4[83]	GTACATCGACATCGTTAACGGCA	biotin labeled
5[665]	ATACCACCATCAGTGAGGCCAAACCGTTGTAGCAA	biotin labeled
4[251]	AACGCCAAAAGGCGGATGGCTTA	biotin labeled
5[119]	CATAATATTCCGTAATGGGATCCGTGCATCTGCCA	external labeling
3[98]	GGATAACCTCACAATTTTTGTTA	external labeling

4[125]	GTTTGAGGGGACCTCATTTGCCG	external labeling
4[144]	CGTAAAGGTCACGAAACCAGGCAATAGCACCGCTTCTGGT	external labeling
0[137]	CATCAGCGTCTGGCCTTCCACAGGAACCTGGGG	external labeling
10[121]	GGGCCGGAAGCATAAAGTG	external labeling
11[135]	TAAAGGATTGTATAAGCGCACAAACGACATTAAATGTGAG	external labeling
7[128]	TTCCGAATTGTAAACGTGTCGCCAGCATCGGTGCGGGCCT	external labeling
3[140]	CAATAGGAACGCAAATTAAGCAA	external labeling
7[105]	GAAAGATCGCACTCCAGCCAGCT	external labeling
0[160]	GATAAAAATTTTTAGCCAGCTTT	external labeling
8[153]	TCAGGCTGCGCAACTGTTGGGAA	external labeling
0[118]	CGAGTAACAACCGTTTACCAGTC	external labeling
2[139]	TTCGCGGATTGATTGCTCATTTTTTAAC	external labeling
10[293]	ACCGGATGTTTTTCTTTTCACCA	5' ATTO 647N
10[279]	ACCCAAATGGCAAAAGAATACTCGGAACAGAATCC	5' ATTO 647N
10[286]	ATTCATTAGAGTAATCTTGACGCTGGCT	5' ATTO 647N
10[265]	AACAAAGCTGCTGTAACAACAAGGACGT	5' ATTO 647N
10[272]	TCAACGTTGCGTATTGGGCGCCAGGGTG	5' ATTO 647N

Supplementary References

1. Derr, N. D. *et al.* Tug-of-war in motor protein ensembles revealed with a programmable DNA origami scaffold. *Science* **338,** 662–665 (2012).
2. Nickels, P. C. *et al.* Molecular force spectroscopy with a DNA origami-based nanoscopic force clamp. *Science* **354,** 305–307 (2016).
3. Vogelsang, J. *et al.* A reducing and oxidizing system minimizes photobleaching and blinking of fluorescent dyes. *Angew. Chem. Int. Ed.* **47,** 5465–5469 (2008).
4. Holzmeister, P., Wünsch, B., Gietl, A. & Tinnefeld, P. Single-molecule photophysics of dark quenchers as non-fluorescent FRET acceptors. *Photochem. Photobiol. Sci.* **13,** 853–858 (2014).
5. Weston, K. D. *et al.* Measuring the Number of Independent Emitters in Single-Molecule Fluorescence Images and Trajectories Using Coincident Photons. *Anal. Chem.* **74,** 5342–5349 (2002).
6. Stangl, T. *et al.* Mesoscopic quantum emitters from deterministic aggregates of conjugated polymers. *Proc. Natl. Acad. Sci.* **112**, E5560-E5566 (2015).
7. Eder, T. *et al.* Switching between H- and J-type electronic coupling in single conjugated polymer aggregates. *Nat. Commun.* **8**, 1641 (2017).
8. Morawska, P. O. *et al.* Side-Chain Influence on the Mass Density and Refractive Index of Polyfluorenes and Star-Shaped Oligofluorene Truxenes. *J. Phys. Chem. C* **119**, 22102-22107 (2015).
9. Manzano, D. A short introduction to the Lindblad master equation. *AIP Advances.* **10**, 025106 (2020).
10. Fischer, K. A. *et al.* Dynamical modeling of pulsed two-photon interference. *New J. Phys.* **18**, 113053 (2016).
